# The benefits of participatory methodologies to develop effective community dialogue in the context of a microbicide trial feasibility study in Mwanza, Tanzania

**DOI:** 10.1186/1471-2458-7-133

**Published:** 2007-07-02

**Authors:** Andrew Vallely, Charles Shagi, Stella Kasindi, Nicola Desmond, Shelley Lees, Betty Chiduo, Richard Hayes, Caroline Allen, David Ross, the Microbicides Development Programme

**Affiliations:** 1 London School of Hygiene and Tropical Medicine, Keppel Street, London, WC1E 7HT, UK; 2 African Medical and Research Foundation, Lake Zone Programme, PO Box 1482, Mwanza, Tanzania; 3 National Institute for Medical Research, Mwanza Centre, PO Box 1462, Mwanza, Tanzania; 4 Medical Research Council, Social & Public Health Sciences Unit, University of Glasgow, Glasgow G12 8RZ, UK

## Abstract

**Background:**

During a microbicide trial feasibility study among women at high-risk of HIV and sexually transmitted infections in Mwanza, northern Tanzania we used participatory research tools to facilitate open dialogue and partnership between researchers and study participants.

**Methods:**

A community-based sexual and reproductive health service was established in ten city wards. Wards were divided into seventy-eight geographical clusters, representatives at cluster and ward level elected and a city-level Community Advisory Committee (CAC) with representatives from each ward established. Workshops and community meetings at ward and city-level were conducted to explore project-related concerns using tools adapted from participatory learning and action techniques such as listing, scoring, ranking, chapatti diagrams and pair-wise matrices.

**Results:**

Key issues identified included beliefs that blood specimens were being sold for witchcraft purposes; worries about specula not being clean; inadequacy of transport allowances; and delays in reporting laboratory test results to participants. To date, the project has responded by inviting members of the CAC to visit the laboratory to observe how blood and genital specimens are prepared; demonstrated the use of the autoclave to community representatives; raised reimbursement levels; introduced HIV rapid testing in the clinic; and streamlined laboratory reporting procedures.

**Conclusion:**

Participatory techniques were instrumental in promoting meaningful dialogue between the research team, study participants and community representatives in Mwanza, allowing researchers and community representatives to gain a shared understanding of project-related priority areas for intervention.

## Background

Community leaders and development professionals have used participatory techniques such as rapid rural appraisal (RRA), participatory rural appraisal (PRA) and participatory learning and action (PLA) since the 1980's in developing countries to design and implement locally appropriate community-based interventions [1,2]. These approaches (commonly now referred to as PLA) use tools such as participatory mapping, community transect walks, seasonal calendars, daily time-use analysis, chapatti or Venn diagrams, scoring, ranking and matrices to facilitate dialogue among and between community members, local stakeholders and development workers and aim to put communities at the forefront of their own development [2-5]. PLA has grown to include techniques and approaches from applied anthropology (such as rapid ethnographic assessment), agro-ecosystems research and participatory action research [2,6-9]. Rapid participatory appraisal is an allied approach which has been used extensively in the health and social sector in industrialised countries, particularly for needs assessment [10-13] and more recently in participatory monitoring and evaluation [14,15].

One reason for the development and spread of these techniques is said to have been a realisation among development workers, researchers and community members of the limitations of 'traditional' approaches to community-based appraisal and research in which surveys were administered by outsiders with limited involvement of communities either at the research design, data interpretation or project implementation stages [16]. An understanding that partnership and effective dialogue are essential for the success of community-based health interventions is not new, however: one of the earliest documented examples being that of the Framingham Community Health and Tuberculosis Demonstration which ran from 1917 to 1924 [17]. Participatory planning in public health is now well established both in developed [18] and developing [19] countries but the use of such techniques to support communities participating in clinical trials remains in its infancy.

The development of candidate vaginal microbicides and vaccines for HIV prevention has brought new challenges [20-24]. In many countries, phase III efficacy and safety trials are feasible only among disadvantaged communities at high-risk of HIV and STIs, where it is difficult for outsiders to meaningfully engage with community members or to develop locally appropriate systems of community liaison. Concepts of community representation, participation, partnership and dialogue may be difficult to apply in such settings [3] and distrust between potential participants and external researchers may be high [25]. For example, Nyamathi et al (2004) used participatory techniques to build trust and effective partnerships with low-income, homeless minority populations in Los Angeles in preparation for a future preventive HIV vaccine trial. They concluded that understanding community perceptions regarding participation in HIV prevention trials is essential if appropriate informed consent procedures, eligibility criteria and participant incentives are to be developed and that respecting community values and priorities is key to ensuring adequate enrolment, cohort retention and the overall success of the trial.

During a feasibility study in preparation for the Microbicide Development Programme's (MDP) phase III efficacy and safety trial of the candidate vaginal microbicide PRO2000/5, a community liaison system was established at the MDP Mwanza site in the Lake Victoria region of Tanzania. The study was conducted among women working in food outlets and recreational facilities in ten administrative wards within Mwanza City. Some women in this occupational group are known to periodically supplement their income through transactional sex and although not necessarily perceived as commercial sex workers within the broader community [26-29], are nonetheless at increased risk of STIs and HIV infection [30-32].

The overall objectives of the feasibility study were to: test the feasibility of recruiting and retaining sufficient numbers of women for a later phase III HIV prevention trial; assess baseline HIV/STI prevalence, HIV incidence and pregnancy rates; investigate methods for improving the reliability and validity of reported sexual behaviour data including condom use and vaginal hygiene practices; and investigate the acceptability of vaginal microbicide gels among women and their sexual partners. The study was implemented through an established collaborative research partnership between the African Medical and Research Foundation (AMREF), the London School of Hygiene and Tropical Medicine (LSHTM) and the National Institute for Medical Research (NIMR) in Mwanza. These institutions have jointly implemented a variety of highly successful research and health development projects in the Lake Zone region over the last 15 years including a large community-based STI intervention trial, an antenatal syphilis screening and treatment project, the Mine Health Project and other initiatives [33]. The collaboration is widely recognized for providing high quality clinical care in Tanzania.

This paper describes how effective partnerships and community dialogue were fostered by an approach based on the ideology underlying PLA and how participatory methods were used to facilitate greater understanding between study participants in the community and the research team.

## Methods

### Study area and population

In March 2002, community-based project fieldworkers identified and visited all food outlets and recreational facilities in ten administrative wards in Mwanza City, classified facilities according to the following predetermined criteria, and recorded the number of women working at each facility. We defined a guesthouse/hotel as any facility with guest beds, a bar as a place primarily to drink, a restaurant as a place primarily to eat, a *kilabu *as a place where locally brewed beer (*pombe*) is bought and consumed on site, and a *mamalishe *as a local food-vending site where women gather at lunchtime to serve food that has been partly prepared at home during the morning. *Mamalishe *is also the term widely used to describe the women who work at such sites. Ward maps were developed to show the approximate location of facilities within each ward relative to local landmarks. An estimated 2,494 women were working in 953 food and recreational facilities. The baseline socio-demographic and other characteristics of women who participated in the study are described elsewhere [32].

Having obtained written consent (signature or thumbprint) from facility owners and managers, trained community-based fieldworkers conducted mobilisation meetings with women at each facility to provide potential participants with information about the study. Weekly community-based reproductive health clinics were established within selected guesthouses or hotels in ten wards by October 2002. Free reproductive health services, including syndromic management of sexually transmitted infections (STIs), family planning, health education and voluntary HIV counselling and testing (VCT) were provided. Women were encouraged to use the free drop-in reproductive health service at any time between their scheduled three-monthly clinic appointments.

A team of social scientists carried out focus group discussions (FGDs) and in-depth interviews (IDIs) between March and May 2003 to explore participant and clinic staff perceptions of the range and quality of clinical services provided by the project. Three FGDs were conducted with clinic staff and six with study participants. Fifteen IDIs were conducted with women who irregularly attended or who had dropped out of the study to explore reasons for non-attendance and impressions of the study team and services provided.

Ethical clearance was obtained from the Medical Research Coordinating Committee in Tanzania and the London School of Hygiene and Tropical Medicine UK. Written informed consent was obtained from all participants prior to enrolment.

### Development and structure of the community liaison system

**Figure 1:**
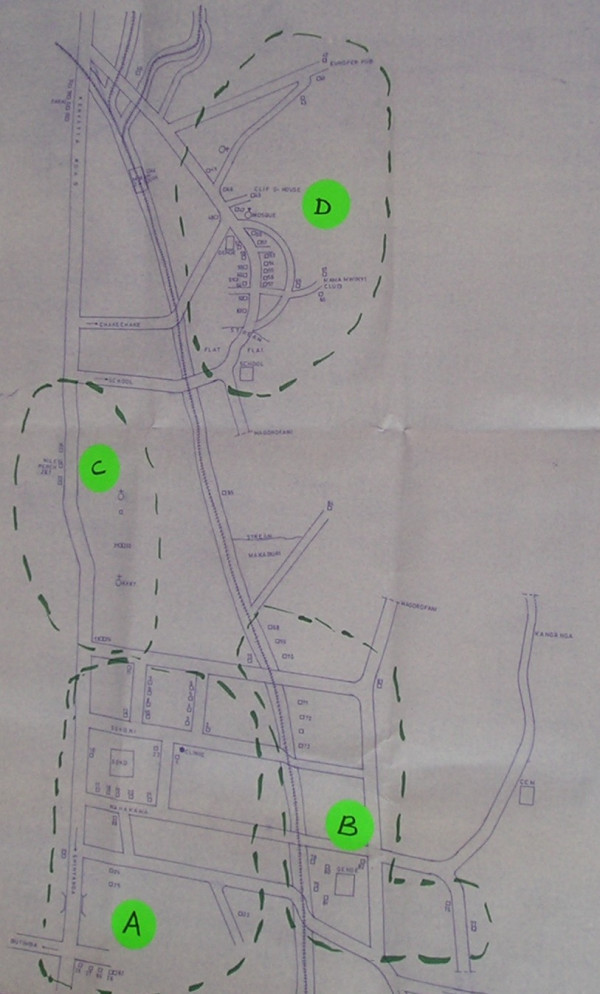
Ward map showing facility clusters.

The mapping exercise showed that certain types of workplace facilities tend to cluster together naturally within wards: informal food vendors (*mamalishe*) and traditional bars (*vilabu*) tended to be located close to one another in less affluent areas; modern bars, restaurants, guesthouses and hotels tended to be located in more affluent areas. This posed difficulties in designing a participatory, representative system of liaison between study participants and researchers. During preliminary community meetings with participants we became aware that differences in income level, age and other factors meant that women working as *mamalishe *or in *vilabu *might feel uncomfortable or embarrassed to approach a community representative working in a modern bar or hotel and vice versa. We therefore decided to build on the natural clustering observed in the mapping exercise and to use this as the basis for our community liaison system in Mwanza. Two cluster types were defined (Table 1) and seventy-eight discrete geographical clusters, each comprising on average around seven individual facilities, drawn on project ward maps (Figure 1).

**Table 1:** Cluster types

**Cluster category**	**Characteristics of facilities within the cluster**	**Characteristics of women working at facilities**
[A]*Mamalishe*/*Kilabu*	• low-income, self-employed businesses	• older women with 'traditional' appearance e.g. wear long dress, headscarf, wrap-around skirt (*khanga*)
	• open air, makeshift, temporary structure (e.g. bamboo walls, packed mud floor, grass thatch roof)	• younger women working at the facility tend to be relatives and receive little or no pay
	• facilities typically owned and managed by women	
	• generally located away from main streets in less affluent areas	
[B] Bars, hotels, guesthouses, restaurants, other	• staff receive a salary plus tips from customers	• younger women with more 'Western' appearance e.g. wear jeans, T-shirt
	• established businesses in permanent structures (e.g. concrete walls, floor; iron or tiled roof)	
	• typically owned by men; women employed as barmaids, waitresses, cleaners, receptionists and in food preparation	
	• generally situated in more affluent areas of town	

A further series of meetings allowed us to gather information from participants on suitable criteria for the selection of community representatives. Key concerns were that representatives should be able to maintain confidentiality, be open and approachable, willing to attend meetings and to represent others. Seventy-eight cluster representatives and eighteen ward level representatives were elected in a process facilitated by the project's Community Liaison Officer (CLO). A high level of coverage was achieved: of the 1573 women who enrolled in the feasibility study, 1418 (90%) worked in a facility represented through the project community liaison system [34].

We held a series of training workshops at facilities in each ward to ensure that both researchers and representatives were clear about the role the community liaison system would play in the development of the project and in assisting preparations for the phase III microbicide trial. Representatives were asked to keep a notebook to record any concerns brought to them by study participants to help them provide feedback at monthly ward meetings chaired by the CLO. Ward representatives were invited to participate in the site-level Community Advisory Committee (*Kamatii ya Ushauri ya Jamii*), established in October 2003.

We developed a logical framework [35] to ensure community activities remained focussed and consistent with the overall objectives of the feasibility study. Indicators were designed to measure the effectiveness of the community liaison system in terms of participation, representation and ability to capture, prioritise and respond to key project-related community concerns.

### Methods of data collection

Between September 2003 and March 2004, a series of one-day community workshops were held at ward and site-level and participatory techniques used to gain a deeper understanding of project-related concerns prevalent among study participants.

#### Listing, scoring, ranking

**Figure 2:**
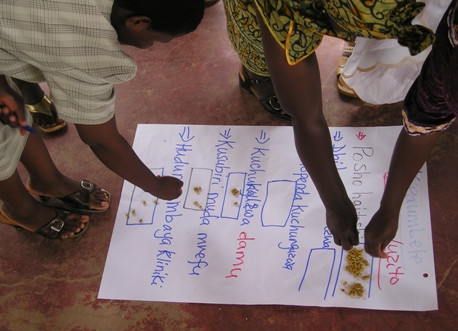
Scoring.

Community representatives in facilitated groups of 12–16 participants were asked to describe key issues and concerns related to the feasibility study, based on their own observations and notes made in their notebooks following conversations with other study participants. Each issue was listed on flipchart paper by the facilitator (the project Community Liaison Officer, CS). Once the list was complete, the sheet was placed on the floor. Each participant was given twelve dried seeds, asked to place these next to the issues that they felt were most important and advised to distribute seeds in any way they wished e.g. five seeds next to a key issue of concern, three to the second most important; one or two seeds next to 5–6 issues of equal importance (Figure 2). Once all participants had voted in this way, a summary score was obtained for each issue and a new list in order of priority drawn up, presented and discussed.

#### Chapati diagramming

**Figure 3:**
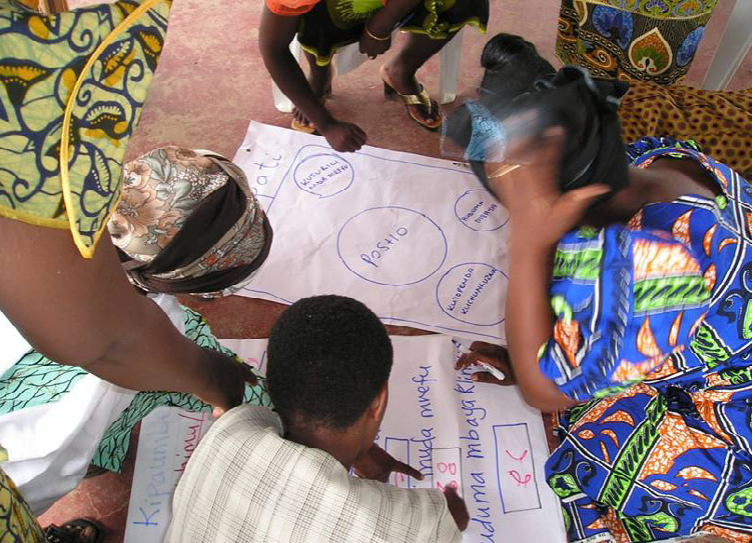
Developing a chapatti diagram.

Participants were asked to break into smaller groups of 4–6 and to draw circles on flipchart paper to represent the relative importance of each of the top 5–10 priority issues relative to one another (Figure 3). This technique is typically used to explore relationships between institutions and individuals within institutions but was employed here to promote further discussion about the initial priority ranking developed. The size and location of each circle relative to the centre of the diagram was used to indicate the relative importance of each issue. Each group fed back their discussions to the broader group and chapatti diagrams were compared and discussed.

#### Pair-wise ranking

**Figure 4:**
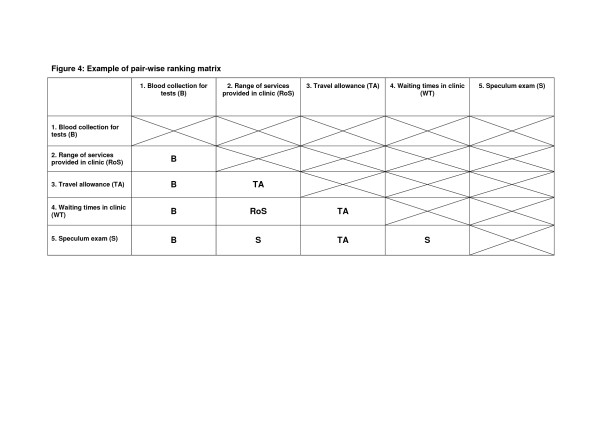
Example of pair-wise ranking matrix.

Next, a pair-wise matrix was drawn up to allow key priority issues to be compared in pairs. This allowed the group to debate and if necessary vote by a show of hands which of the two issues being discussed was the more important. For example, in Figure 4 the issue of blood collection was compared with range of services, travel allowance and other issues in turn. Blood collection was felt the more important issue in each case as indicated by the 'B' in the matrix where the column and row issues bisect. Similarly, travel allowance was felt more important than either speculum examination or clinic waiting times.

### Developing appropriate responses and monitoring impact

Having developed a priority list of concerns, appropriate project responses were developed over the next 12–15 months (April 2004 – July 2005) in partnership with the Community Advisory Committee (CAC) through discussion at site-level meetings. The appropriateness and impact of the response chosen was gauged through informal feedback from our network of local community representatives, discussions at CAC meetings and with clinic staff at weekly project staff meetings.

## Results

### Identifying and responding to priority concerns

FGDs and IDIs conducted in March 2003 revealed that the following issues were of concern to study participants:

• Blood collection

• Confidentiality

• Speculum examination

• Time spent at the clinic

• Travel allowance level

• Stigma

• Care for HIV positive women

It was difficult from this information alone to conceptualise how the various issues might be ranked in order of perceived priority among study participants and which issues the project should therefore attempt to address first. In the community workshops, similar themes emerged but by using the participatory techniques described above, it was possible to explore key issues in depth and to develop a ranked list of priorities (Table 2). This summary list was developed in a site-level participatory workshop following a series of earlier workshops at ward level. Differences between perceived priorities in individual wards were discussed and reviewed during the pair-wise ranking process and a final ranked list of priorities agreed. This became the basis of an action plan for project-community partnerships designed to make study implementation more transparent, acceptable and locally appropriate.

**Table 2:** List of project-related concerns by order of priority

**Issue (ranked in order of priority)**	***Comments made during participatory workshops***
1. Blood taking	*'why do you take so much blood every time?'* *'blood might fall into the wrong hands and be sold for witchcraft purposes'*
2. Allowances	*'we are losing money when we come to clinic'*
3. Speculum examinations	*'how do we know the speculum is safe [clean]?'*
4. Range of services provided	*'why can't we bring our children to the clinic when they are sick?'* *'you should treat malaria and fever in children'* *'our men don't like to go to hospital [for STI treatment] – why can't we bring them to the clinic'*
5. Clinic waiting times	*'sometimes we wait a long time to be seen'*
6. Laboratory test results	*'some tests take a long time to come back'* *'I went [to another clinic] and got my result straight away after I had already waited a long time for my result from your clinic'*
7. Care and support for women who are HIV positive	*'how can you help me if I am/become HIV positive?'*
8. Stigma	*'people [in the community] laugh at us for coming to the clinic - - they think we must be HIV positive'* *'people think that clinics are only for people who are HIV positive'*
9. Treatment issues	*'why are the drugs you give us so strong? If we take them we cannot work properly'*
10. Confidentiality	*'my photograph might appear in the newspaper with my HIV result'*

#### Blood specimen collection

During participatory workshops this issue was consistently identified as the most important project-related concern among study participants. Informal feedback from community representatives had already alerted us about rumours (*uzushi*) in the community that blood was being collected in order for it to be sold for witchcraft purposes, an issue which also featured prominently in the earlier focus group discussions, the data from which have been reported in detail elsewhere [36]. Representatives reported that the volume of blood collected (10 mL/participant) was also of concern to some participants, who considered it a large amount.

We responded to these issues using a combination of approaches. First, members of the CAC were invited to see how blood specimens are handled following collection in the clinic and submission to the laboratory at the National Institute for Medical Research (NIMR), Mwanza. Ward representatives participated in a half-day visit to NIMR when they visited different parts of the laboratory in small groups and observed how blood is stored, centrifuged and the separated serum used to test for HIV, HSV and syphilis. Participants were able to see first hand that all the blood collected is either tested or stored at the laboratory and that relatively large amounts of blood are needed. A demonstration on how genital specimens are prepared and examined was provided and participants invited to use the microscope to see pathogens for themselves. CAC members declared the trip a great success and asked that regular visits be organised in future. At cluster level, project fieldworkers conducted community meetings and met with individual participants to provide information about the handling of blood specimens. Cluster representatives were advised to discuss the NIMR visit with their ward representative and to counter any *uzushi *heard by giving details of this trip. Finally, a flipchart for community mobilisation was developed by the project in collaboration with the CAC in September 2004 and included a section specifically on *uzushi *to try and tackle this and other issues head on. Fieldworkers used the flipchart in facility-based meetings attended by 8–10 potential participants, who were then invited to attend a project clinic if interested. Pictures of blood being collected in the clinic have now been incorporated into a revised site flipchart currently in use during the main trial in Mwanza.

#### Travel allowance

The travel allowance (*posho*) provided to compensate participants for costs incurred travelling to clinic were felt to be inappropriately low by women participating in community workshops. Women working as *mamalishe*, which are essentially single-handed self-employed businesses, complained that they were actually losing money when they come to clinic because they miss out on several hours selling food and that the *posho *(at that time TSH 500 or approx. USD 0.50) was not enough to compensate for this. This was less of an issue for bar workers who typically work in the evenings and are paid either a small salary and/or a commission based on the number of drinks sold. Information from FGDs suggested that most *mamalishe *earn around TSH 4000 – 6000 per day. The project team and the CAC debated whether it would be appropriate to increase allowances only for *mamalishe*. Clinic staff felt that the logistics of introducing differential rates would be too onerous and both staff and community representatives felt such a system would be widely perceived as unfair by participants and community stakeholders. Allowance levels for all participants were therefore raised to TSH 1000 in January 2004 and after further review and discussion with the CAC, raised again to TSH 3000 in July 2004 prior to a small pilot study designed to test the acceptability of study procedures and vaginal gel in preparation for the start of the main phase III trial. Favourable feedback from pilot study participants and at subsequent CAC meetings suggests that this response was appropriate and welcomed by participants. Allowance levels are being reviewed in participatory workshops every six-months during the phase III trial when other types of support will also be considered e.g. project staff have suggested providing tea and a light snack (cake or chapatti) for women on arrival in clinic.

#### Speculum examination

Participatory techniques highlighted concerns about the safety of the speculum examination and specifically, women's worries that it would not be possible for specula to be adequately cleaned before each examination. In FGDs and IDIs, participants expressed concerns about the frequency of speculum examination (conducted at enrolment and six-monthly), did not see the value of genital examination if they were asymptomatic and also raised the issue of speculum cleanliness and safety.

A multifaceted approach was again adopted. Cluster and ward representatives were invited to observe the operation of the project autoclave and were surprised at the length of time required (longer than expected), the amount of steam and heat produced and the use of indicator tape to confirm successful operation e.g. *'...now we know they must be clean...no germs *(vijidudu)*could have survived that!' *At community mobilisation meetings, project fieldworkers stressed that specula were cleaned using a steam autoclave, explained the need for regular clinical examinations even in those without symptoms, passed round an speculum for participants to handle and responded to questions. Informal feedback from community representatives suggests that these activities have helped improve understanding of these issues. Data from IDIs conducted between December 2004 and January 2005 following completion of the pilot study support this assertion. Pictures of a speculum have now been incorporated into a revised site flipchart currently in use during the MDP301 clinical trial in Mwanza. We will also be exploring the acceptability of single-use plastic specula in the first six-months of the phase III trial with participants, cluster representatives and CAC members.

#### Range of services provided

In participatory workshops, community representatives felt that the project was providing high quality clinical services overall but that the range of services could be improved. Specifically, the project was criticised for failing to provide services for participants' children and male sexual partners. Project staff subsequently explained at cluster, ward and site-level meetings and on an individual basis in clinics that it is not currently possible to provide such an extended range of services: the project was designed to provide community-based sexual and reproductive health services to women working in food and recreational facilities following earlier research in Mwanza which highlighted the difficulties such women face when attempting to access established local services [37,38]. Following discussion with local health care providers in late 2005, referral networks for male partners have been revised, strengthened and streamlined, an initiative that has been received positively by members of the CAC. At the time of writing, it was considered too early to gauge the more widespread acceptability of this approach among study participants in the phase III cohort. Work is ongoing to strengthen referral systems for infants and children.

#### Waiting times to be seen in the clinic

Some community representatives felt waiting times in the clinic were too long. *Mamalishe *tend to attend clinic late in the afternoon once the main business of the day (lunch orders; 12.00 to 2.00 pm) is over. In wards with a high proportion of *mamalishe *this often results in longer waiting times because clinics are typically busy by early afternoon and women arriving at these times may join a short backlog of women waiting to be seen. Women working in other types of facilities had a different view. For example, bar workers who typically work evening/night shifts and attend clinics in the early part of the morning before other women arrive, felt clinic waiting times were acceptable. Women from all facility types felt the time required to complete study procedures was overlong and asked if visits could be shortened in any way.

Discussions between clinical staff, community-based fieldworkers, community representatives and study participants have attempted to improve understanding and promote dialogue about the reasons why clinic waiting times can sometimes be lengthy e.g. clinical review and counselling can take up more time with some participants than others according to the nature of the consultation or the complexity of issues being discussed. Encouraging women to attend clinics at scheduled times and to try and arrive earlier in the day has largely been unsuccessful. Periodic time and motion studies have identified bottlenecks in clinic flow and allowed clinic procedures to be streamlined. The phase III trial currently underway in Mwanza, and in other MDP sites in Africa, includes 'long' clinical follow-up visits (approx. 60 minutes) and 'short' gel collection visits (approx. 30 minutes). Participants are given information about the visit schedule and the reasons behind it at facility-based mobilisation meetings and during informed consent procedures. CAC members have welcomed these approaches and we will be exploring these issues further in future participatory workshops.

#### Laboratory test results

The project was commended for the quality of clinical care provided but community representatives felt they waited too long for the results of some diagnostic tests, particularly HIV and syphilis results. Laboratory-based HIV ELISA assays were used in feasibility because of extensive experience with these tests in previous research conducted in Mwanza and the absence of national guidelines on the use of HIV rapid tests at that time (May 2002). It was not possible to provide a same-day results service using the ELISA assays; participants received their results during post-test counselling at a subsequent visit 1–2 weeks later. In participatory workshops, community representatives complained that other local VCT service providers were able to provide same day HIV results [using HIV rapid tests] and felt dissatisfied with our service in comparison. Project counsellors expressed similar views and urged a review of study test procedures.

Syphilis serology was also by laboratory-based assay with a similar turnaround time for results. In practice, difficulties tracing participants (to advise them to return to clinic for treatment) and inefficient reporting systems (based on monthly lists of results by study number) meant that many participants only received their results after several weeks, which we agreed was unacceptable.

The project responded to these concerns in September 2004 by introducing clinic-based HIV rapid tests, which by now were being rolled-out nationally; by improving procedures for tracing participants to their homes and workplaces; and by streamlining procedures for reporting syphilis and other test results. Clear timelines for reporting laboratory results to the clinics have been established. At a CAC meeting in December 2004, representatives felt that participants had readily accepted the revised procedures and we were advised to continue these into the main trial.

#### Stigma

Participants' experiences of stigmatisation due to clinic attendance was raised at participatory workshops, CAC and cluster meetings. Representatives reported that verbal abuse and petty discrimination are common experiences for women working in bars and other facilities in Mwanza and that some participants had experienced an increase in stigmatisation once their participation in the study became known within the wider community. Other women feared that if their participation became known they might risk losing their job, or be verbally or physically abused by their husbands or partners.

To date it has not been possible to address key factors likely to be associated with stigma in this community, such as misconceptions; fear of infection through casual contact; moral and religious attitudes to promiscuity [39], but sensitive, targeted community mobilisation has allowed specific cases to be tackled. For example, one bar owner was sacking employees if he discovered that they were attending our clinics. He feared that it would affect his business if clients found out, since they would assume his workers were HIV positive. Following discussion with project fieldworkers, he now actively encourages his staff to attend so they may receive free reproductive health care and allows his facility to be used for regular ward-level meetings.

The project has been careful not to exacerbate stigma and has therefore always adopted a low-key presence in the community. For example, mobilisation activities are conducted in small groups at facility-level rather than by general community meetings or through announcements in the mass media. As support has grown among community stakeholders, a feeling that this approach may have been over-cautious has developed both within the project and among CAC members. For example, when project T-shirts were introduced for participants at the end of 2004, rather than increase stigma (as we initially feared), anecdotal evidence from community representatives who wore them suggests that they helped promote debate, local interest and greater awareness of our work. In partnership with the CAC we plan to introduce participatory theatre in selected wards during the main trial as part of an expanded community mobilisation strategy and by incorporating elements from the HIV-Stigma Toolkit developed by The CHANGE Project [40], we hope to dispel rumours, prevent misconceptions and help to alleviate discrimination experienced by women participating in the trial.

#### Care and support for HIV positive women

This issue was raised in participatory workshops and at all levels of the community liaison system in Mwanza. During feasibility we established links with local non-governmental and community-based organisations providing care and support for people living with HIV/AIDS and built a referral network for women to address a variety of needs including counselling, legal advice, home-based care and spiritual support. An agreement between the project and the major local public health provider of antiretroviral therapy has allowed direct referrals during the main trial: women diagnosed as HIV positive at screening or who seroconvert during the course of the trial are referred for clinical assessment, CD4 count and if appropriate, antiretroviral therapy. Feedback from the Mwanza City HIV/AIDS Management Committee is that these initiatives are appropriate in this context and an example of good practice for other local development projects. Participatory workshops and IDIs in 2006/07 will assess client and service provider perceptions.

#### Treatment issues

Community representatives were concerned that some of the drugs prescribed in the clinic are 'too strong' (Table 2), especially bar workers who are often offered alcohol by men as a prelude to transactional sex but who are advised by clinic staff not to drink if they are taking antibiotics. Misunderstandings regarding drug regimens have been discussed with community representatives and clinic staff advised to counsel clients carefully about the importance of completing entire courses of antibiotic treatment and avoiding sex during treatment for STIs.

## Discussion

Community advisory boards, liaison committees and stakeholder groups have been used in a variety of research settings to ensure the acceptability and appropriateness of study procedures, channels of communication and media messages to the local study population and the broader community from which it is drawn [41-45]. FGDs, IDIs and other 'formal' social science methods have been used to assess participant and community perceptions of many such interventions, for example in Uganda [46] and Ethiopia [47]. We used participatory tools in an attempt to better understand project-related concerns and priorities from the perspective of study participants and community representatives and found them a useful adjunct to conventional qualitative methods with important additional benefits. Similar benefits have been reported from both developed and developing country settings [5,48]. Firstly, we were able to develop joint understanding and agreement as to which issues should be considered important and prioritised for review and action. It was made clear from the outset that some issues were either beyond the scope of the project or would be extremely difficult to tackle e.g. providing services for children. Community representatives at participatory workshops and CAC meetings welcomed this open, transparent approach even if they did not agree with the reasons why such change was not possible. Secondly, a cyclical process (namely: issue collection; prioritisation; review; developing and implementing a response; feedback) was facilitated and involved the CAC together with the research team at each key step. This has allowed draft pictorial flipcharts, proposed clinic procedural changes, strategies for cluster-based mobilisation activities and other issues to be agreed in consultation with women representing study participants prior to their introduction. Perceptions of a placebo gel and views on how to explain the concept of 'placebo' clearly to potential participants in the main trial have also been sought and useful insights gained. Third, as a result of this approach, researchers have over time developed a relationship of mutual trust and understanding with cluster and ward representatives that has in turn lead to a shared belief that as far as possible, the clinical trial will be conducted as a partnership between the research team and the community. Inequalities and differences in power and influence are openly recognised by both the CAC and the research team so that this is unlikely to ever be more than an unequal partnership but all agree we are at least entering the main trial together within the context of an open and effective relationship. As researchers, we also feel that we have been granted a form of 'community consent' to return to bars, guesthouses and other facilities to conduct mobilisation activities for the main trial.

Participatory research tools complemented formal research methods and increased our confidence in the validity of the data collected. In addition, subtle differences in emphasis observed in some areas (e.g. safety and cleanliness of speculum vs. frequency and need for repeated examinations) would likely have been missed using either formal or informal methods alone, hindering the development of appropriate responses to these issues.

Are there other benefits to such approaches in clinical trials? Representative, participatory liaison systems provide an informal mechanism for rapid, early feedback of trial-related issues to the research team; particularly those systems that ask study participants to take the lead rather than secondary community stakeholders e.g. representatives of non-governmental or community-based organisations. In HIV treatment and prevention trials, and indeed in any trial involving new (unlicensed) investigational products, informal early warning of possible adverse events, poor acceptability of study product and trial procedures is extremely helpful in maintaining participant safety and in ensuring the ethical conduct and successful conclusion of the trial. Emerging rumours, misconceptions and misinformation can also be addressed effectively and appropriately before they adversely affect participant recruitment or cohort retention. This approach allows researchers to more easily access and develop meaningful dialogue with people in stigmatised, vulnerable high-risk groups, which are often those with the greatest burden of HIV and the most feasible study populations in which to conduct HIV prevention trials from the epidemiological perspective [21-25,30].

Given the apparent benefits, what are the challenges and limitations of this approach? Effective structures and systems of representation are paramount and key to understanding who 'community representatives' actually represent. It is also important for researchers and representatives to develop joint understanding of the meaning of 'community' in order to help ensure adequate, appropriate representation. Community in this context can be conceptualised as participants within the study cohort only; members of the occupational group from which they were recruited; or more broadly as all women living in selected wards in Mwanza City. Definitions of community and the design, development and implementation of the cluster-based system used in Mwanza for community representation will be discussed in detail elsewhere (Shagi C; in draft). In brief, 90% of women within the research cohort worked within a designated geographical cluster and 77% of a random sample of 85 participants from 20/78 clusters told independent interviewers that they knew their cluster representatives and had interacted with her at least once in the past to discuss a project-related concern. Around 60% of the 85 women had interacted with their representative in the four weeks prior to the interview. A risk in adopting the approach we advocate is that without structured, representative systems for community liaison, data generated in participatory workshops might be misleading and attempting to respond to the issues raised, counter-productive.

Another possible limitation is that by asking participants to carry out activities together there is a risk of a lack of independence of responses. For example, during issue-scoring later contributors may tend to follow the opinions of the first few who put seeds down. We used a variety of complementary tools to facilitate open debate and to try and ensure such effects were minimised. By conducting a series of workshops at different levels within the liaison system we were also able to build an understanding of key themes from different community perspectives and to further reduce this potential risk.

Measuring the impact of these activities and processes also represents a challenge. A participatory management cycle has been set in motion following our initial series of workshops which we feel requires the development of participatory monitoring and evaluation tools to fully explore measures of success from the community perspective. Communities and development professionals have in a variety of settings compared diagrams, maps and other tools at different time points and carried out joint analysis with community members as to the meaning and implications of their findings[49]. In a series of workshops to be carried out during the first six months of the start of the main trial in Mwanza we will be exploring similar iterative approaches using matrices, timelines, chapatti diagrams and other tools.

## Conclusion

This is the first time to our knowledge that PLA-type tools have been reported in this context. We believe that participatory techniques represent a useful adjunct to formal research methods and that they are essential in their own right in fostering open dialogue, trust and understanding between researchers, study participants and community representatives in preparation for HIV prevention trials. These techniques may be especially valuable for reaching vulnerable, stigmatised high-risk groups in developing countries.

## Competing interests

The author(s) declare that they have no competing interests.

## Authors' contributions

AV conceived, designed and coordinated the use of participatory research approaches in this setting (with CS), conducted the literature review and drafted the manuscript.

CS conceived, designed and coordinated the use of participatory research approaches in this setting (with AV), conducted literature searches and book reviews and helped draft the manuscript.

SK participated in the design and coordination of the study, conducted a situational analysis (with BC, AV) and helped draft the manuscript.

ND participated in the design and coordination of the study, coordinated in-depth interviews and focus group discussions (with BC), and helped draft the manuscript.

SL participated in the design and coordination of the study and helped draft the manuscript.

BC participated in the design and coordination of the study, conducted a situational analysis (with SK, AV), coordinated and conducted in-depth interviews and focus group discussions (with ND), and helped draft the manuscript.

RH participated in the design and coordination of the study and helped draft the manuscript.

CA participated in the design and coordination of the study, assisted ND and BC in the design of qualitative research instruments and helped draft the manuscript.

DR participated in the design and coordination of the study, assisted AV, CS in the community liaison system design phase and helped draft the manuscript.

All authors have read and approved the final manuscript.

## Pre-publication history

The pre-publication history for this paper can be accessed here:


